# Phase separation of NELFE modulates chromatin accessibility to promote dichotomous signaling pathways in hepatocellular carcinoma

**DOI:** 10.21203/rs.3.rs-5843408/v1

**Published:** 2025-04-25

**Authors:** Hien Dang, Alvaro Lucci, Anna Barry, Victoria Johnson, Brittany Ruiz, Laura Reynolds, Adam Wojnar, Kasonde Chewe, Yotsawat Pomyen, Christoph Eckert, Kai Zhang, Jason Hill, Ashesh Shah, Adam Bodzin, Elda Grabocka, Matthias Gaida, Nicolas Fawzi

**Affiliations:** Thomas Jefferson University; Thomas Jefferson University; Thomas Jefferson University; Brown University; Thomas Jefferson University; Thomas Jefferson University; Thomas Jefferson University; Thomas Jefferson University; Thomas Jefferson University; University Medical Center Mainz; Thomas Jefferson University; Thomas Jefferson University; Thomas Jefferson University; Thomas Jefferson University; University Medical Center Mainz; Brown University

## Abstract

Biomolecular condensates partition various cellular processes including transcription, DNA repair, and RNA metabolism. We report NELFE, a member of the Negative Elongation Factor complex required for Polymerase II (Pol II) pausing, forms distinct foci mediated by two low complexity sequences. We show NELFE is oncogenic in hepatocellular carcinoma (HCC) by undergoing liquid-liquid phase separation (LLPS) with SMARCB1 to modulate chromatin accessibility to downregulate pro-apoptotic genes through Pol II pausing while activating pro-growth signals to promote HCC progression. Our work highlights the importance of NELFE LLPS as a mechanism of chromatin accessibility to regulate both paused and non-paused genes to drive tumorigenesis in hepatocellular carcinoma.

## Introduction

A key characteristic of cancer is the significant transcriptomic changes that serve as disease markers and reflect traits that enhance tumor evolution. Some transcriptomic alterations are stable and consistent across various cancers, linked to genetic changes that provide a survival advantage, while others are dynamic and context-dependent. The stable genetic changes are associated with early tumor evolution events, while the dynamic changes indicate cellular plasticity—the ability of cells to alter their identity beyond normal developmental processes to promote tissue homeostasis. This plasticity enables cancer cells to adapt and evolve, making both stable and dynamic transcriptomic changes vital for cancer cell survival. However, the mechanisms regulating these coordinated events remain unclear.

Liquid-liquid phase separation (LLPS) is a key mechanism that regulates transcription by compartmentalizing proteins and nucleic acids into membraneless organelles known as biomolecular condensates. Driven by intrinsically disordered regions (IDRs) or low complexity sequences (LCS), LLPS facilitates weak multivalent interactions that promote cooperative demixing^1^. These biomolecular condensates play a crucial role in controlling biochemical reactions related to chromatin organization, genome stability, transcription regulation, stress responses, and RNA/protein degradation^2–3^. In the cytoplasm, stress granules (SGs) are essential for cell survival, aiding in translation regulation, mRNA storage, and cell signaling by rapidly assembling and dispersing in response to stress. In the nucleus, biomolecular condensates such as nucleoli, cajal bodies, nuclear speckles, paraspeckles, and promyelocytic leukemia (PML) are important in genome regulation affecting ribosome biogenesis, RNA synthesis, storage, processing, and chromatin organization(1). More recent work has shown RNA Polymerase II (Pol II) can form “gene-body condensates” composed of the transcription machinery, including transcription factors, and coactivators such as the Mediator complex to ensure gene activation(2, 3). It remains unclear how nuclear condensates facilitate dynamic transcription in cancer.

Pol II promoter-proximal pausing is a highly regulated process that allows for rapid synchronous gene activation important for biological processes such as stress(4). DRB sensitivity inducing factor (DSIF) and the Negative Elongation Factor (NELF) complex, which consists of NELFA, NELFB, NELFC/D, and NELFE, mediates Pol II pausing by localizing Pol II at the proximal-promoter region after transcribing 20–60 nucleotides (nt) of RNA(5). Pol II’s release into active transcription or elongation, requires positive elongation factor b (p-TEFb), a part of the cyclin-dependent kinase 9 (CDK9) and Cyclin T complex(6). More recent work showed NELF, through the NELFA IDR, form stress-inducible condensates that drive the coordinated downregulation of transcription, likely an important canonical transcriptional response to stress(7). Interestingly, NELF-depleted cells show a reduction of paused Pol II at promoters, with few genes upregulated, and many genes downregulated at the mRNA level^12,13^. In line with these finding, a current study implicates that NELF acts distinctly from the Pol II pausing^14^. Additionally, we also showed that loss of NELFE can lead to both increase and decreased gene expression important for hepatocellular carcinoma progression(8). It remains unknown how or why NELF depletion (either NELFA or NELFE loss) downregulates so many genes distinctly from Pol II pausing.

Recent works have implicated dysregulated LLPS affecting chromatin accessibility as a potential malignant driver of cancer biology(9). For example, the ARID1A gene is mutated in more than 8% of all cancers, which is often found within the IDR regions(10, 11). In addition to mutations affecting LLPS function, gene fusions can cause a gain in LLPS function. In acute myeloid leukemia (AML), the NUP98-HOXA9 fusion protein can mediate homotypic and heterotypic LLPS to modulate transcription of malignant transformation(12). Notably, NUP98 fusion oncoproteins occur in more than 5% of all AMLs(13). Abnormal LLPS can also disrupt signal transduction pathways, contributing to cancer development as in the case of the accumulation of glycogen in liver cancer cells activating oncogenic YAP signaling, promoting the survival and transformation of hepatocellular carcinoma (HCC) cells(14).

In this work, we demonstrate that oncogenic NELFE undergo LLPS to modulate chromatin accessibility(8). We also dissect the mechanism by which NELFE LLPS with the chromatin modulator, SMARCB1, to enhance Pol II pausing of pro-apoptotic genes, while distinctly enhancing chromatin accessibility of pro-survival genes in HCC. Our work highlights the importance of LLPS modulation of the chromatin of both paused and non-paused genes as a regulatory mechanism important for adapting to the imbalance in cancer cells.

## Results

### NELFE undergoes liquid-liquid phase separation.

**Figure 1:**
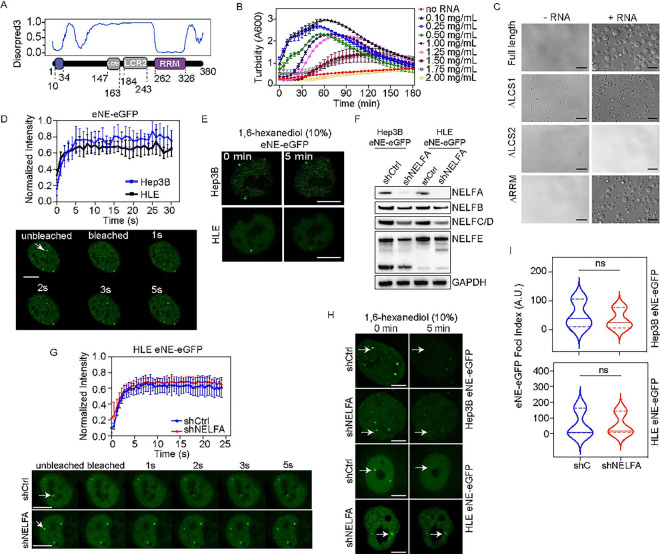
NELFE undergoes phase separation independent of NELF. (**A**) Disordpred3 schematic of NELFE protein. (**B**) Turbidity assay with increasing concentrations of RNA at various time points. (**C**) NELFE liquid droplets observed by differential interference contrast (DIC) microscopy at high concentrations with or without RNA in high salt concentrations. Scale bars are 20uM. (**D**) (Top) Fluorescent recovery after photobleaching (FRAP) analyses of NELFE foci in eNE-eGFP cells. n=30 cells, data represents mean±SD. (Bottom) Confocal still images of Hep3B eNE-eGFP cells imaged before and after FRAP. Images are 63X and scale bar are 10 uM. Arrows point to NELFE foci. (**E**) Live cell imaging of eNE-eGFP cells before and after 1,6-hexanediol treatment. Images are at 63X and scale bar is 10 uM. (**F**) Immunoblot of NELF complex proteins in eNE-eGFP cells with shRNA mediated NELFA or Ctrl knockdown. (**G**) (Top) FRAP analyses over time of NELFE foci in HLE eNE-eGFP cells treated with shCtrl or shNELFA. n=30 cells, data represents mean±SD. (Bottom) Still confocal images of HLE eNE-eGFP cells before and after bleaching. Images are 63X and scale bar are 10 uM. Arrows point to NELFE foci. (**H**) Live cell imaging of eNE-eGFP cells treated with shCtrl or shNELFA for 72 h followed by 1,6-hexanediol treatment. Images are at 63X and scale bar is 10 uM. (**I**) Violin plots with median (solid line), dotted line represents quartiles of foci index (ratio of foci/cell) (n=20 cells, ns=not significant, Student’s t-test). See also Figure S1.

NELFE forms distinct foci in the nucleus which resemble condensates or nuclear bodies known to regulate chromatin accessibility(15). Confocal immunofluorescence analyses of NELFE showed that the foci did not co-localize with different nuclear bodies, including Coilin, HP1, PML, and SC35 (**Figure S1A and S1B**)(16). These results suggest that NELFE foci are distinctly different than nuclear bodies. Thus, we hypothesize that NELFE foci are potentially membrane-less organelles that can modulate transcriptional changes through LLPS. Specifically, NELFE has two low complexity sequences (LCS) and one RNA Recognition Motif (RRM) but does not have a canonical intrinsically disordered region (IDR), which is known to drive LLPS. Accordingly, DISOPRED3 predictions show that the NELFE LCS regions are highly disordered (Figure 1A), suggesting NELFE may have the ability to undergo LLPS. To determine whether NELFE can LLPS we used several approaches. First, we cloned the full length NELFE fused with GFP at the C’ terminus into a pJ4M backbone and performed a liquid droplet assay in vitro (Figure 1B)*(17)*. We performed turbidity assays with purified recombinant NELFE and increasing concentrations of RNA to measure NELFE’s ability to form liquid droplets and found NELFE formed liquid droplets in an RNA-dependent manner (Figures 1C **and S1C)**. Next, we deleted the LCS1, LCS2, or RRM and observed both the LCS1 and LCS2 mutants abolished liquid droplet formation, whereas the deletion of the RRM had minimal effect in high salt concentration(Figures 1C). Furthermore, when NELFE-mGFP was overexpressed in Hep3B cells, full length NELFE-mGFP and the ΔRRM-mGFP mutant formed large nuclear foci, whereas the ΔLCS1 mutant foci appeared smaller (**Figure S1D**). Consistent with the *in vitro* assay, the deletion of the LCS2 abolished foci formation. Second, we determined the dynamic and reversible nature of NELFE foci endogenously by employing CRISPR/Cas9 mediated homology-directed repair (HDR) to tag the NELFE locus with enhanced Green Fluorescent Protein (eGFP) at the C’ terminus in Hep3B and Huh1 HCC cell lines. In both cell lines, we were able to successfully knockin eGFP (eNELFE-eGFP) as evident by immunoblotting (**Figure S1E**). Immunofluorescence analyses of two different NELFE antibodies (epitope in the N’ terminus region and recombinant full length NELFE) showed co-localization of eGFP and NELFE antibodies (**Figure S1F**). Using these knockin models, we employed fluorescence recovery after photo-bleaching (FRAP) to determine the dynamics of eNELFE-eGFP, we called eNE-eGFP foci. Photobleaching of the eNE-eGFP foci showed they recovered within seconds of photo-bleaching (Figure 1D). Moreover, 1,6-hexanediol (1,6-hex) treatment, an alcohol that dissolves condensates by interfering with weak hydrophobic interactions, showed eNE-eGFP foci are sensitive to 1,6-hex within five minutes (Figure 1E) (18). Together, these data suggest that eNE-eGFP foci are dynamic biomolecular condensates.

To determine whether eNE-eGFP foci is distinct from NELF, we genetically knocked down NELFA in both HCC knockin cell models. As expected, loss of NELFA reduced the expression of NELFE and NELF proteins (Figure 1F) but had no significant effect on recovery after photo-bleaching (Figures 1G **and S1G**). The eNE-eGFP remained sensitive to 1,6-hex treatment after 5 min (Figure 1H). Although the knockdown of NELFA had no impact on the dynamics of the NELFE foci in both HCC cell models, there were quantitatively less eNE-eGFP foci. Additionally, violin plot revealed shNELFA treatment reduced the number of large eNE-eGFP foci (Figure 1I). These data are consistent with current work which has shown that NELF LLPS requires NELFA(7). However, our work supports the notion that while NELFA is important for NELF LLPS, NELFE is required for LLPS, which may occur distinctly from the NELF complex.

### NELFE modulates chromatin accessibility.

**Figure 2:**
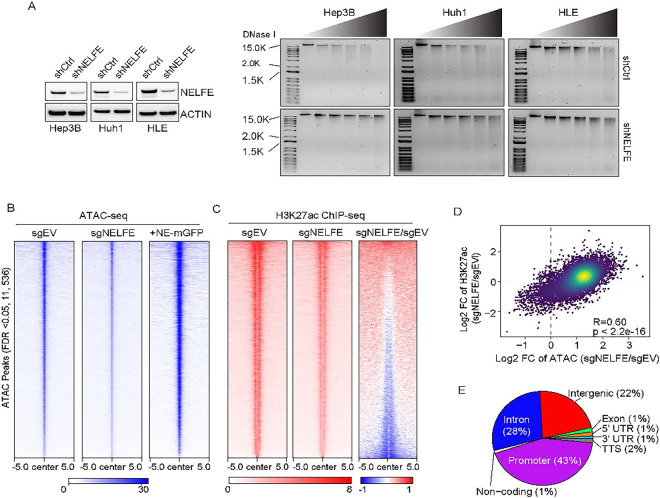
NELFE modulates chromatin accessibility. (**A**) (Left) Immunoblot of NELFE in Hep3B, Huh1 and HLE cells with shRNA mediated NELFE knockdown. (Right) Agarose gel of HCC cells transduced with shCtrl or shNELFE virus for 72 h and treated with DNASe I (0, 15, 20, 25, 30, 35 U). (**B**) Heatmap of 11,536 ATAC consensus peaks in CRISPR/Cas9 mediated knockout of NELFE in Hep3B cells followed by NE-mGFP overexpression. (**C**) H3K27ac ChIP peaks in order of decreasing signal. For the heatmap of fold changes (sgNELFE/sgCtrl), the color bars depict log2 values. (**D**) Spearman correlation scatterplot of log2FC between sgNELFE/sgCtrl of Hep3B CRISPR cells. (**E**) Pie chart analysis of accessible regions affected by NELFE knockout. See also Figure S2.

To determine the effect of NELFE on the chromatin, we performed DNAse I digestion assay by subjecting genomic DNA of HCC cells treated with short hairpin mediated knockdown of NELFE (shNELFE) or a scramble control (shCtrl) to increasing concentrations of DNAse I. We observed reduced fragmentation of DNA across three HCC cell lines treated with shNELFE compared to shCtrl (Figure 2A). Moreover, the overexpression of NE-mGFP rescued these effects in both CRISPR-mediated NELFE loss HCC cell lines, including Hep3B and HLE (**Figures S2A and S2B**). Next, we coupled the assay for transposase accessible chromatin followed by next-generation sequencing (ATAC-seq) with H3K27ac ChIP-sequencing to identify changes in chromatin accessibility that are transcriptionally active in the Hep3B CRISPR NELFE knockout (NELFE KO) model. We identified 7,923 genes (11,536 ATAC/H3K27ac consensus peaks t (FDR < 0.05)) across all replicates. Knockout of NELFE resulted in a pronounced decrease in chromatin accessibility, which was rescued upon NE-mGFP overexpression (Figure 2B). Consequently, these same regions saw a significant reduction in H3K27ac signal (Figure 2C), which was highly correlated with ATAC-seq peaks (Figure 2D), suggesting NELFE predominantly affects the accessibility of active transcription sites, largely located within the promoter regions (Figure 2E).

### NELFE modulation of chromatin accessibility affects both paused and non-paused genes.

**Figure 3:**
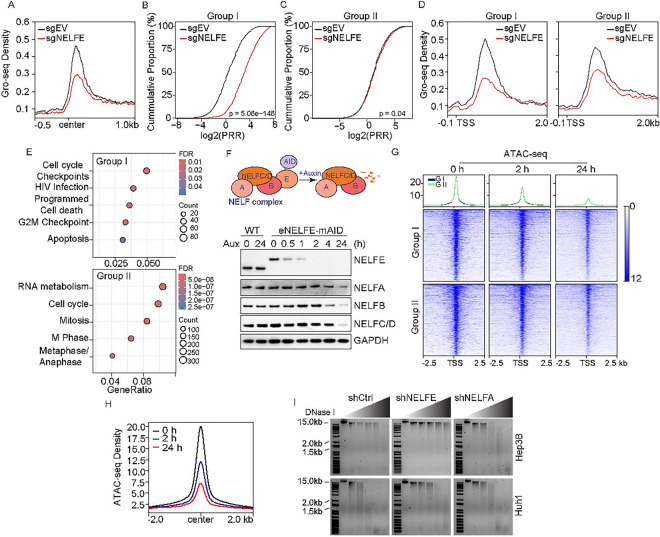
NELFE depletion effects both paused and non-paused genes. (**A**) Metaplot of all (5,974) high, low to moderately paused genes in sgNELFE and sgEV showing NELFE depletion has a stronger effect on highly paused genes. (**B-C**) ECDF plots of promoter release ratio (PRR) for highly paused (Group I) and low to moderately paused (Group II) genes. (**D**) Metaplots of Group I and Group II GRO-seq reads from −100 bp to 2kB around the TSS. (**E**) Over-representation analysis of Group I and Group II genes (FDR<0.05). (**F**) (Top) Schematic of eNELFE-mAID system. (Bottom) Immunoblot of the NELF complex (NELF-A, B, C/D, and E) in Hep3B eNELFE-mAID and parental (WT) cells treated with 1uM 5-Ph-IAA at various time points. (**G**) Heatmaps of Group I and Group II. Rows are sorted by decreasing ATAC-seq intensity signal. (**H**) Centered metaplot of 9,731 ATAC-seq peaks at 0, 2, and 24 h of 5-Ph-IAA treatment. (**I**) Agarose gel of Hep3B and Huh1 cells after 72 h of shRNA mediated knockdown of NELFE (shNELFE), NELFA (shNELFA) or control (shCrtl). gDNA from each group was treated with DNASe I (0, 15, 20, 25, 30, 35 U). See also Figure S3.

NELFE is an important member of the NELF complex, which is involved in Pol II pausing, a mechanism by which cells control and synchronize transcription(5). Moreover, NELF-induced Pol II pausing has been shown to facilitate chromatin accessibility and NELF-mediated stalling of Pol II can enhance gene expression by blocking the promoter-proximal nucleosome(8, 19–21). To determine whether NELFE’s effect on chromatin accessibility is through NELF, we performed global nuclear run-on sequencing (GRO-Seq) to map the distribution of engaged Pol II genome-wide in the Hep3B NELFE KO cells. The overlap between ATAC, H3K27ac ChIP-seq and Gro-seq data yielded 7,918 consensus peaks (FDR <0.05) or 7,305 genes. Metaplot of these genes showed reduced engaged Pol II near the promoter and NELF complex protein expression, including NELFA, NELFB and NELFC/D is reduced (Figures 3A **and S3A**). Pol II pausing at promoters can range from low to moderately high occupancy and occurs predominantly in actively transcribed genes(22). To identify highly paused genes affected by NELFE, we calculated the pausing release ratio (PRR, the ratio of Pol II density in gene bodies over Pol II density at the promoter region) (**Figure S3B**), which estimates the release of promoter-proximal paused Pol II into productive elongation(23, 24). As expected, empirical cumulative distribution function (ECDF) plot showed loss of NELFE increased elongation (**Figure S3C**). To identify specific genes affected by Pol II pausing, we classified highly affected and moderately to low affected Pol II paused genes based on the PRR fold change. We found 1,626 genes with a >1.5 PRR_FC (log2 [PRR_FC] > 0.585), which we referred to as Group I (Pol II Paused) and 3,598 genes with a <1.5 PRR_FC (− 0.585 < log2 [PRR_FC] < 0.585), which we referred as Group II (Others) (Table S1). ECDF plot and metaplot showed group I genes are more affected than group II genes with higher levels of engaged Pol II near promoters (Figures 3B–3D). Using over-representation analyses (ORA, hypergeometric p-value <0.05), we identified five unique pathways associated with Group I or Group II (Figure 3E). Specifically, we found genes involved in cell cycle checkpoints, programmed cell death, and apoptosis were enriched in Group I, whereas genes involved in cell cycle were enriched in Group II.

To determine whether NELFE affects chromatin accessibility distinct from the NELF complex, we employed the mini-auxin-inducible degron (mAID) version 2 system to rapidly degrade NELFE using CRISPR/Cas9-mediated HDR (Figure 3F, top)(25). The endogenous NELFE gene is tagged with the mAID cassette at the C’ terminus in Hep3B cells that constitutively express the AID adaptor protein OsTIR1. In this model system, eNELFE-mAID protein was predominantly degraded within 2 h of 5-Ph-IAA (a derivative of Indole-3-acetic acid (IAA) treatment but had no effect on the parental line (Figure 3F, bottom). Specifically, NELFA, NELFB, and NELFC/D remained stable up to 4 h of 5 Ph-IAA treatment, but disappeared at later time points, consistent with previous studies(26). Based on these results, we chose 2 h of 5-Ph-IAA treatment for further experiments to ensure loss of NELFE while minimizing the effects of other NELF complex proteins on chromatin accessibility.

Using this system, we first determined the effects of NELFE degradation on chromatin accessibility using the DNAse I assay. We found DNAse I digestion was efficient at low concentration in HCC cells treated with 5-Ph-IAA at 0 h but not at 2 or 24 h. Moreover, there was less DNA fragmentation at 2 h compared to 24 h, consistent with previous reports that NELF contributes to chromatin accessibility (**Figure S3D**)(19, 27). To map DNA regions affected by NELFE-mAID degradation, we performed ATAC-seq in the same time points (**Figure S3E**). We reason that at 2 h, any observed alterations in genome-wide chromatin accessibility are induced by NELFE distinct from NELF compared to 0 h; whereas changes observed at 24 h are additive effects of both. We overlapped peaks from the NELFE-mAID with the NELFE KO and identified 11,130 consensus peaks (FDR < 0.05) or 7,792 genes. Metaplot of these genes revealed a slight reduction in signal from 0 to 2 h for both groups and a significant reduction in signal from 2 to 24 h (Figure 3G). Moreover, we found no observable difference in signal across the time points between the two groups (Figure 3H). Finally, we determined whether the NELF complex requires NELFE to affect chromatin accessibly by targeting NELFA, which directly interacts with Pol II, via shRNA. Immunoblot and DNASe I digestion assay in Hep3B and Huh1 cell lines showed loss of NELFA reduced the expression of NELF proteins, including NELFE, within the NELF complex (**Figure S3F**). Although the loss of NELFA showed reduced DNA fragmentation, genomic DNA in HCC cells with NELFE knockdown required higher DNASe I concentration (Figure 3I). Altogether, our data indicate that NELFE directly affects the chromatin accessibility and gene subsequent expression of both paused and non-paused genes.

### Liquid-liquid phase separation promotes HCC progression.

**Figure 4:**
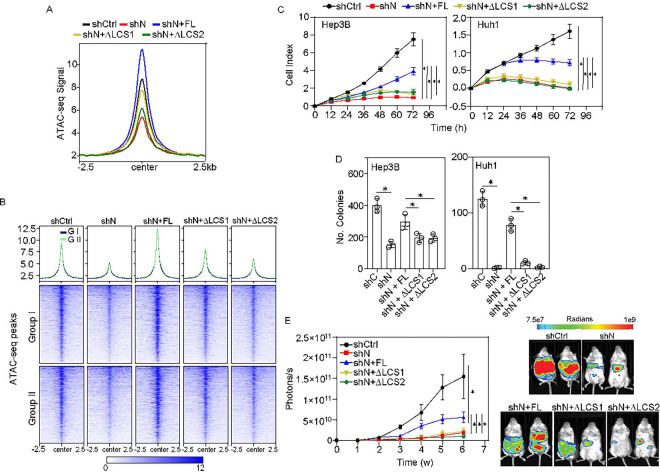
Liquid-liquid phase separation promotes HCC progression. (**A**) Centered metaplot of 9,731 ATAC peaks after shRNA mediated NELFE knockdown and rescued with specified mutants. (**B**) Heatmap of 9,731 ATAC peaks after knocked down NELFE using shRNA and rescued with various mutants. Rows are sorted by decreasing ATAC intensity signal. (**C-D**) Cell proliferation and colony formation assays, respectively in Hep3B and Huh1 cells of NELFE knockdown with shRNA followed by specific mutant overexpression (*p-value < 0.001, Student’s t-test, data mean±SD). (**E**) (left) Mean signal of tumor growth from NELFE knocked down and subsequently rescued with mutants for 6 weeks. (Right) Bioluminescence of NOD/SCID mice at six weeks. (*p-value < 0.01 from One-way ANOVA with Tukey’s post hoc test, data represents mean±SEM, n=6 mice/group). See also Figure S4.

To investigate whether NELFE LLPS affected chromatin accessibility, we knocked down NELFE using shRNA (shNELFE) and rescued various mutants of NELFE, including full length, ΔLCS1 or ΔLCS2 (**Figure S4A**). DNAse I digestion assay showed the mutants ΔLCS1 or ΔLCS2 reduced the fragmentation of DNA compared to shCtrl or full-length (**Figure S4B**). Next, we performed ATAC-seq to determine the genome-wide effect on chromatin accessibility by the mutants. Metaplot and heatmaps showed neither ΔLCS1 nor ΔLCS2 mutants were able to fully rescue accessible regions compared to full-length NELFE in Hep3B cells with shNELFE (Figure 4). However, there was a distinctly larger effect in the ΔLCS2 mutant, possibly because the loss of the LCS2 completely disrupts NELFE LLPS, whereas the LCS1 only partially affects it. When we separated the regions into group I or group II, we saw no difference in signal, indicating that NELFE LLPS affects chromatin accessibility of both paused and non-paused genes (Figure 4B). Additionally, neither the LCS1 nor LCS2 mutants were able to rescue the expression of NELF complex compared to full length (**Figure S4C**).

Next, we determined the effects of NELFE LLPS on HCC progression via cell proliferation and colony formation assays. Consistent with our previous work, shRNA-mediated knockdown of NELFE reduced cell proliferation in Hep3B, HLE, and Huh1 cells. Using a low multiplicity of infection (MOI) since high concentrations of NELFE can cause cell death, we were able to rescue cell growth upon NE-mGFP (full length) overexpression (Figures 4C **and S4D-S4E**). However, the ΔLCS1 or ΔLCS2 mutants were not able to rescue cell proliferation or colony formation compared to full-length, whereas there was no significant difference between ΔLCS1 or ΔLCS2 (Figures 4D **and S4F**). Intrahepatic injection of Huh1 cells with NELFE knockdown showed that tumor progression was significantly reduced (Figure 4E **and Figure S4G-S4H**). Consistent with the *in vitro* data, neither ΔLCS1 nor ΔLCS2 mutants were able to rescue tumor growth compared to full length *in vivo*, indicating that NELFE LLPS is important for HCC growth. All together, these data show that while the ΔLCS2 mutant completely inhibits NELFE LLPS, LCS1-induced LLPS modulation of chromatin accessibility is necessary for NELFE’s tumor promoting function.

### DLCS1m is important for HCC.

**Figure 5:**
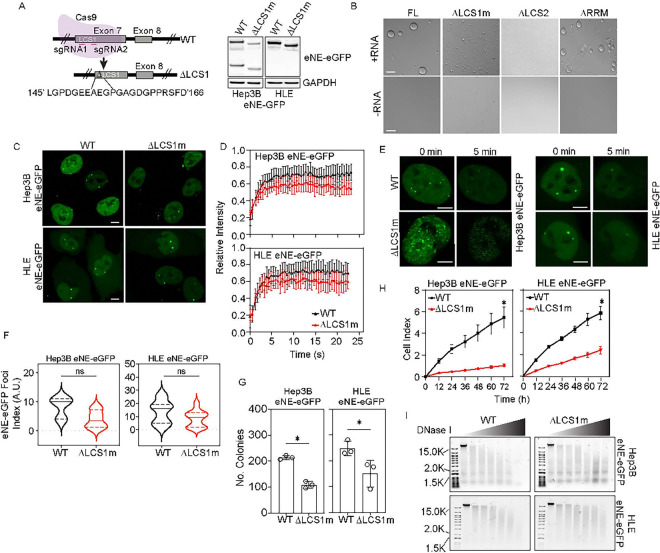
NELFE LCS1-induced LLPS is important for HCC. (**A**) (left) Schematic of CRISPR/Cas9 genome editing of the eNEΔLCS1-eGFP mutant model. (Right) Immunoblot of WT or eNEΔLCS1-eGFP mutant. (**B**) ΔLCS1 liquid droplets observed by DIC with or without RNA in high salt concentrations. Scale bars are 20uM. (**C**) Live cell images of both Hep3B and HLE WT or eNEΔLCS1-eGFP mutants (63X, scale bar at 10 uM), (**D**) FRAP analyses over time of NELFE foci (n=30 cells, data represents mean±SD), (**E**) Confocal still images of 1,6-hexanediol treatment (63X and scale bar at 10 uM). Arrows point to droplets. (**F**) Violin plots of foci index (ratio of foci/cell). n=20 cells, dashed lines represent quartiles, and solid line represents the median (ns=not significant, Student’s t-test). (**G-H)** colony formation and cell proliferation assays, respectively in WT or eNEΔLCS1-eGFP mutant cells (*p-value < 0.05, Student’s t-test, data represents mean±SD). (**I**) Agarose gel of gDNA from each group treated with DNASe I (0, 15, 20, 25, 30, 35, 40 U). See also Figure S5.

To further investigate the role of LCS1 LLPS in HCC, we employed CRISPR/Cas9 to genomically delete the LCS1 region of the NELFE locus using two-gRNA in the eNE-eGFP knockin cell models (Figure 5A, left)(28). Although we tested multiple gRNAs to genomically edit the exact aa143 -163 region, we were only successful at deleting the aa145–166 region we call ΔLCSm. Further confirmation of the genetic alteration showed a shift in NELFE expression in eNEΔLCS1m-eGFP mutant compared to wildtype (WT) (Figure 5A, right). Immunoblot analyses showed the eNEΔLCS1m-eGFP mutant had decreased levels of NELF complex protein expression, including NELFA, B, and C/D (**Figure S5A**). *In vitro* droplet assay showed loss of aa145–166 reduced liquid droplet formation with the addition of RNA, indicating the two amino acids at the N’ terminus and three additional amino acids at the C’ terminus had no significant effect on LLPS (Figure 5B). Consistent with our *in vitro* observations, confocal microscopy analyses revealed ΔLCS1m formed more, smaller foci compared to WT (Figure 5C). Furthermore, the eNEΔLCS1m-eGFP foci have a slower recovery time after photobleaching (Figure 5D) but remained sensitive to 1,6-hex treatment (Figure 5E). Additionally, foci analysis via confocal microscopy of the eNEΔLCS1m-eGFP cells revealed there were smaller and less foci compared to WT (Figure 5F). Furthermore, eNEΔLCS1m-eGFP mutants grew significantly slower and formed less colonies compared to WT (Figure 5G–H). To characterize the eNEΔLCS1m-eGFP mutant’s effect on chromatin accessibility, we used DNAse I assay and observed reduced fragmentation of genomic DNA in the ΔLCS1m mutants (Figure 5I). Together, these data indicate that the LCS1m is sufficient for the tumor promoting function of NELFE in HCC, which could be attributed by both NELFE and NELF.

### NELFE LLPS with SMARCB1 to modulate chromatin accessibility.

**Figure 6:**
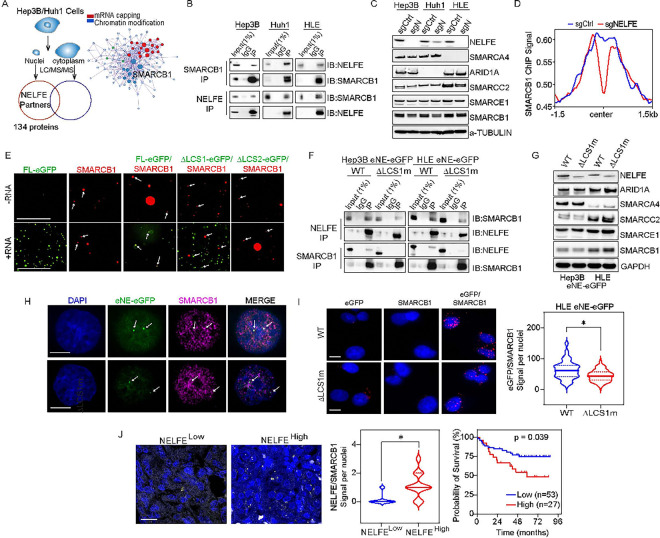
NELFE LLPS with SMARCB1 to modulate chromatin accessibility (**A**) Schematic of mass spectrometry of NELFE from nucleus of Hep3B and Huh1 cells and Metascape analyses of 134 nuclear proteins. (**B**) Co-immunoprecipitation immunoblot of SMARCB1 and NELFE in HCC cell lines. (**C**) Immunoblotting of SWI-SNF complex protein expression in HCC cells with NELFE genomically altered via CRISPR/Cas9. (**D**) Centered metaplot of 11,536 peaks in SMARCB1 ChIP-seq from Hep3B cells. (**E**) Colocalization fluorescence microscopy images of liquid droplets formed by NELFE-eGFP and/or SMARCB1 in the presence or absence of RNA. SMARCB1 was tagged with Alexa Fluor 594-labeled. Scale bar is 100uM.Arrows point to droplets. (**F**) Immunoblot of co-immunoprecipitation of SMARCB1 and NELFE in WT and eNEDLCS1-eGFP cells. (**G**) Immunoblot of SWI/SNF proteins in WT and eNEDLCS1-eGFP cell lines. (**H**) Confocal images of immunofluorescence of NELFE and SMARCB1 co-localization. Images are at 63X, and scale bar is 10 uM. Arrows point to NELFE foci. (**I**) Proximity ligation assay on HLE eNEDLCS1-eGFP cells. (Left) Representative images showing the merged red (probe signal) and blue (DAPI) channels. Images are at 40X, and scale bar is 10 uM. (Right) Quantification of foci/nuclei in cells treated with SMARCB1, GFP, or SMARCB1/GFP antibody. Dashed lines represent quartiles, and darker line represents the median (*p-value < 0.05, Student’s t-test, error bar is SEM). (**J**) Proximity ligation assay on HCC samples. (Top) Quantification of foci/nuclei in cells treated with SMARCB1, NELFE, or SMARCB1/NELFE antibody. Dashed lines represent quartiles, and darker line represents the median (*p-value < 0.05, Student’s t-test, error bar is SEM). (Bottom) Representative images showing the merged yellow (probe signal) and blue (DAPI) channels. Images are at 40X, and scale bar is 10 uM. See also Figure S6.

We hypothesize that NELFE undergoes LLPS with chromatin modulators to regulate accessibility. First, we performed immunoprecipitation of NELFE followed by mass spectrometry of fractionated nuclear lysates of two HCC cell lines, Hep3B and Huh1, to identify potential NELFE binding partners. Selecting for proteins found only in the nucleus but not the cytoplasm, we identified 134 proteins (Figure 6 **and Table S2**). Metascape analyses revealed chromatin regulators and mRNA capping as two of the key mechanisms potentially involved in NELFE nuclear function (Figure 6A). Two key chromatin modulators were significantly enriched, including SMARCB1 and SUZ12. Specifically, we focused on SMARCB1, a core subunit of the SWI/SNF (BAF) complex involved in chromatin remodeling because of its recent link to Pol II pausing and HCC(29, 30). In HCC, SMARCB1 is rarely mutated and is highly expressed; yet its oncogenic role remains elusive. (29). To confirm the SMARCB1/NELFE interaction, we performed co-immunoprecipitation (Co-IP) and observed SMARCB1 immunoprecipitated NELFE and conversely across three HCC cell lines (Figure 6B).

To assess the effects of NELFE loss on the SWI/SNF complex,we investigated the protein expression of ARID1A, SMARCC2, SMARCE1, and SMARCA4 (BRG1) in the NELFE KO CRISPR cell models. NELFE loss across three HCC cell lines showed no significant change in protein expression of the SWI/SNF proteins. However, SMARCB1 expression was slightly reduced (Figure 6C). In comparing the genome-wide targeting of SMARCB1 in Hep3B cells, we found NELFE KO had no substantial effect on SMARCB1 interaction with the chromatin (**Figure S6A**). However, when we examined SMARCB1 interaction with the chromatin at NELFE affected sites, SMARCB1 signal was distinctly reduced (Figure 6D). To link SMARCB1 and chromatin accessibility, we performed DNAse I assay. CRISPR/Cas9 mediated SMARCB1 loss showed reduced DNA fragmentation in a concentration dependent matter across two HCC cell lines, Hep3B and HLE (**Figure S6B**).

Next, we determined whether NELFE and SMARCB1 co-phase separate by purifying SMARCB1 and NELFE-GFP recombinant proteins and performed the liquid droplet assay *in vitro* (Figure 6E). As expected, NELFE-eGFP full length formed liquid droplets with RNA, whereas SMARCB1 also formed droplets and aggregates (larger irregular shaped droplets) regardless of RNA addition. Although SMARCB1 and NELFE-eGFP co-phase only with the addition of RNA, not all of NELFE-eGFP droplets co-localized with SMARCB1, possibly due to its propensity to aggregate (Figure 6E). Furthermore, we found that while the NELFE-DLCS1 abolished SMARCB1/NELFE-eGFP co-phase, there remains some NELFE-eGFP or SMARCB1 droplets in the presence of RNA. Finally, as expected the DLCS2 inhibits NELFE-eGFP droplet formation and only SMARCB1 droplets were visible. To further investigate the SMARCB1/NELFE LLPS relationship, we performed co-immunoprecipitation in the eNE-eGFP and ΔLCS1m cells. Immunoblot analyses of the eNEDLCS1-eGFP mutants showed a reduction of SMARCB1 proteins bound to NELFE compared to parental and conversely (Figure 6F). Notably, the expression of SWI/SNF proteins, including SMARCB1 were not affected, indicating that NELFE LLPS, mediated by the LCS1, does not affect the expression of the SWI/SNF complex (Figure 6G). We next leveraged the eNE-eGFP knockin HCC cell models to determine the co-localization of SMARCB1 with NELFE foci *in situ* via confocal microscopy. In both eNE-eGFP HCC cell lines, we found colocalization between eNE-eGFP foci and SMARCB1 (Figures 6H **and S6C**). However, SMARCB1 appeared more proximal to eNEΔLCS1-eGFP mutant foci, suggesting that NELFE LLPS mediated by LCS1 is required for their co-localization. We next performed proximity ligation assay (PLA) to quantify the effect of ΔLCS1 on SMARCB1 and eNE-eGFP interaction *in situ*. In the WT eNE-eGFP cells, SMARCB1 protein was within proximity (<40 nm) of eNE-eGFP foci (Figure 6I **and S6D-E**). In the mutants, there was a significant reduction in proximity of SMARCB1 to the eNE-eGFP foci, indicating that NELFE LLPS with SMARCB1 requires the LCS1. Altogether, our data show NELFE ΔLCS1 abrogates SMARCB1-mediated nucleosome remodeling activity, which can diminish DNA accessibility.

Finally, we tested whether HCC patients with high levels of NELFE also had increased co-localization with SMARCB1. We first performed NELFE IHC analyses on our TJUH cohort (n=80) and Mainz cohort (n=300) using an Allred (>5) method to subgroup patients from NELFE^High^ vs. NELFE^Low^ (**Figure S6F**). We then performed PLA analyses and found NELFE^High^ subgroup had a higher NELFE/SMARCB1 signal per nuclei compared to NELFE^Low^ group, which was also associated with poor survival (Figure 6J **and S6G**).

## DISCUSSION

Here, we report that NELFE can form nuclear condensates with SMARCB1 to modulate chromatin accessibility, which is important for transcriptional suppression of proximal-promoter paused genes and activation of proliferative genes in HCC. By forming condensates at the proximal promoter regions, NELFE can facilitate increased chromatin residence of NELF at highly paused genes, effectively pausing transcription of genes that suppress tumor growth. Additionally, NELFE can mediate chromatin accessibility of pro-survival genes to promote tumorigenesis. Disruption of NELFE LLPS by targeting the LCS1 moderately affects cell recovery after photobleaching and reduces cell proliferation and tumor burden. Together, this modulation is possible through LLPS with SMARCB1, a component of the SWI-SNF chromatin remodeling complex, which plays a crucial role in regulating chromatin dynamics(33, 34). Our work demonstrates a specific LLPS mechanism required for chromatin accessibility of NELFE and NELF of both paused and non-paused genes in HCC.

LLPS is a key mechanism that regulates many aspects of cellular activity which is essential for dynamic chromatin organization and gene regulation(35, 36). Proteins that can undergo LLPS usually contain an IDR, which allows reversible and dynamic multivalent interactions with RNA and/or proteins. Recent work shows that NELF complex can phase separate and condense in response to stress through the IDR of NELFA(14, 25). NELFE does not have a traditional IDR but two LCS domains, which are predicted to be highly disordered. Although an LCS is not a canonical IDR, recent works provide evidence that they can facilitate LLPS(2). In this work, we found that NELFE alone can phase separate with RNA to form liquid droplets independent of the NELF complex *in vitro*. Confirming our *in vitro* results, we found that the deletion of LCS2 completely inhibits droplet formation whereas the deletion of LCS1 moderately affects it. It is likely that the LCS2, which is 69 amino acids in length and consists of RDRD repeats, is required for LLPS and that the LCS1 is important for the specificity of condensate formation. In our cell model system, both LCS regions are important for LLPS; while the deletion of the LCS2 caused cell death and inhibited foci formation, eNEΔLCS1m-eGFP cells showed reduced phase separation, decreased HCC proliferation, colony formation, and tumor progression *in vivo*. A similar effect was observed when we deleted LCS2. However, the LCS2 region contains both the p-TEFb binding and ADP-ribosylation sites, therefore, we cannot completely attribute the aforementioned observed effects to the disruption of phase separation(13, 37, 38). Altogether, these data demonstrate a specific LLPS mechanism required for hepatocarcinogenesis.

NELFE foci are prevalent in HCC cells, which do not require various stressors. Specifically, the knockdown of NELFA did not have a sizeable effect on accessible DNA compared to the loss of NELFE, indicating that NELFE is seminal in maintaining the stability of NELF residence on the chromatin. This may be due to the fact that NELFE has an RRM that is required to bind nascent RNA to mediate NELF-induced Pol II pausing(39). How NELFE affects chromatin accessibility distinctly from NELF is unclear, but one of the possibilities that we approach in this work is that is undergoing LLPS with chromatin modulators, in our case with SMARCB1. Indeed, it has been shown that SMARCB1, acting distinctly from the SWI/SNF complex, acts as a tumor suppressor in rhabdoid tumors by promoting Pol II pausing of MYC target genes(30). However, in rhabdoid tumors SMARCB1 is mutated and nonfunctional, leading to a significant activation of oncogenic signals(40).. Previous work has indicated that SMARCB1 is overexpressed in liver tumors compared to non-tumor and its elevated expression is associated with poor prognosis(31). Interestingly, we observed large SMARCB1 foci in HCC cells, which were also observed in human HCC patient samples (data not shown). Moreover, we found SMARCB1 also formed droplets *in vitro* regardless of RNA, and loss of SMARCB1 had minimal effect on the expression of the SWI/SNF proteins. These data suggest that SMARCB1 can phase separates distinctly from the SWI/SNF complex. Herein, we show that NELFE can phase separate with SMARCB1 and demonstrate that disrupting NELFE LLPS affects their interaction, leading to a loss of co-localization between the two proteins. Although NELFE LLPS mediates chromatin accessibility of some paused genes, strikingly we found that the majority of accessible regions affected were at non-paused genes in HCC.

Cancer development is a complex and multidimensional process that can select for cells resistant to various stressors. It is known that NELF regulation of proximal-promoter Pol II pausing is required for rapid responses to these stressor stimuli including heat shock response, estrogen signaling, and inflammation(16, 41, 42). Moreover, NELF can also regulate transcription distinct from RNA Pol II pausing in response to stress(17). In HCC, more than 90% of all cases occur in the setting of chronic (inflammatory) liver disease with various etiologies including, metabolic diseases (MASLD), alcoholic induced fatty liver disease, or hepatitis B/C infection(43). These chronic inflammatory signals can lead to the selection of stress resistance mechanisms that provide a survival advantage. Thus, as a stress responsive coping mechanism during tumor evolution, biomolecular condensation may facilitate increased NELF residence time at the chromatin to promote transcriptional downregulation of pro-apoptotic and programmed cell death genes(14). Conversely, it is also likely that increased NELF phase separation may provide increased accessibility to cell cycle genes, a key mechanism in cancer cell proliferation. Consequently, our work shows that NELFE foci are distinct biomolecular condensates necessary for chromatin modulation of both paused and non-paused genes. Specifically, our work provides a model whereby NELF-mediated Pol II pausing through NELFE can be selected for tumorigenesis.

## Resource availability

### Lead contact

Further information and requests for resources, reagents, and analysis code should be directed to and will be fulfilled by the lead contract, Hien Dang (hien.dang@jefferson.edu)

### Materials availability

This study did not generate new unique reagents.

### Data and code availability

All sequencing datasets raw fastq files and processed data have been deposited on NCBI GEO (TBD) and are publicly available as of the date of publication.

### All original code is deposited on GitHub.

Any additional information required to reanalyze the data reported in this paper is available from the lead contact upon request.

## Method

### Human Samples

Formalin-fixed paraffin embedded (FFPE) HCC patient tissues were collected from patients with HCC from the years 2018–2020 at the Thomas Jefferson University Hospital in full compliance with the ethical guidelines of the National Institutes of Health (NIH) and with the approval of the Thomas Jefferson University Hospital Institutional Review Boards (IRB Control #20D.138) for the collection and use of human tissues for research.

### Cell Lines

Huh1 and HLE cells were cultured in Dulbecco’s modified Eagle Medium (Thermo) supplemented with 10% fetal bovine serum (FBS), penicillin, streptomycin and L-glutamine. Hep3B cells were cultured in Minimum Essential Medium (Thermo) supplemented with 10% FBS, penicillin, streptomycin and L-glutamine, non-essential amino acids (Thermo) and sodium pyruvate (Thermo). Mycoplasma testing was performed every month on cultured cells using the LookOut Mycoplasma PCR detection kit (Sigma MP0035). All cell lines were authenticated using STR DNA profiling analyses using the Promega PowerPlex^®^ 16 System annually. All cells were kept in a 37°C humidity incubator with 5% CO2. Cells were cultured only up to 15 passages from freeze thaw.

### Animal Study

All animal studies were approved by the Thomas Jefferson University Institutional Animal Care and Use Committee (Protocol #02259). Nine-weeks-old female NOD/SCID mice (NOD.CB17-Prkdcscid/NcrCrl) from The Jackson Laboratory (Bar Harbor, ME). Huh1 cells were stably transfected with the luciferase expression vector at first and then were transfected with lentivirus overexpressing NELFE CDS (full length, ΔLCS1 and ΔLCS2) using an MOI of 0.5. The day after, media was changed, and cells were transduced with shNELFE lentivirus with 8 μg/ml polybrene for 24 hr and incubated overnight. Media was then changed and 2 μg/ml puromycin was supplemented for selection for 3 days. Cells were then trypan blue counted and 1×106 Huh1 cells were suspended with DMEM media (1×10^6^ cells/50 μl) and injected into the left hepatic lobe of NOD/SCID mice using Matrigel as a vehicle. Every week, luciferase signals were measured using IVIS Lumina Series III (PerkinElmer, Waltham, MA) after intraperitoneal injection of 3mg D-Luciferin (PerkinElmer). Luciferase signals were quantified using Aura imaging software (Spectral) At 6 weeks, mice were sacrificed.

### Human Tissue Array IHC and PLA Assay

HCC tissues were obtained with informed consent from patients who underwent radical resection from the Liver and Pancreas Tissue biorepository (2018–2020, Thomas Jefferson University Hospital (TJUH). The studies were approved by the TJUH institutional review board. Tissue microarrays were immunostained with anti-NELFE, anti-SMARCB1, or DAPI and 50% of each tumor-center spot was imaged at 40×. Imaging and analysis were performed blinded.

### Statistical Analysis

Statistical analysis for cell proliferation, colony formation, tumor growth, foci index and granularity per nuclei were done in GraphPad Prism 10 (v10.2.3) and other statistical analysis was done in R (v.4.3.1). For two groups a student’s t-test is performed and for multiple groups, one-way ANOVA with Tukey’s posthoc test. Adjusted p-value is presented when multiple groups are tested. The Kolmogorov-Smirnov (K-S) test was used to compare sample distribution in all empirical distribution functions.

## Statement of Significance

Polymerase Pol II pausing is important for gene regulation and transcriptional control. Here, we show liquid-liquid phase separation of Negative elongation Factor E with SMARCB1 simultaneously promotes increased Pol II pausing at pro-apoptotic and increased chromatin accessibility of pro-survival genes in hepatocellular carcinoma.
